# Inappropriate ICD Shocks in Pediatric and Congenital Heart Disease Patients

**DOI:** 10.19102/icrm.2017.081104

**Published:** 2017-11-15

**Authors:** Jason M. Garnreiter

**Affiliations:** ^1^Department of Pediatrics, Saint Louis University School of Medicine, St. Louis, MO, USA

**Keywords:** Congenital heart disease, implantable cardioverter-defibrillator, inappropriate shock, pediatric

## Abstract

Although implantable cardioverter-defibrillators (ICDs) have proven to be life-saving devices, there are frequent complications associated with their use, especially in the pediatric and congenital heart disease populations. Inappropriate shocks are a particularly frequent complication in these groups. This review discusses the causes and implications of inappropriate ICD shocks, and presents potential interventions that may assist in safely reducing the rates of inappropriate shocks in pediatric and congenital heart disease patients with ICDs.

## Introduction

Since the original description of the use of an implantable cardioverter-defibrillator (ICD) in a human,^1^ the use of ICDs has expanded rapidly. In this time, numerous large, prospective studies involving adult patients have demonstrated that these devices can provide significant survival benefits for patients with a history of a cardiac arrest event (as a secondary prevention method), or in those deemed to be at high risk of experiencing a cardiac arrest event due to underlying cardiovascular disease (as a primary prevention method).^2–7^ Pediatric patients and patients with congenital heart disease (CHD) represent a small minority of ICD recipients, and there have been no prospective randomized trials of ICD use in this heterogeneous population.^8,9^ Still, numerous retrospective studies have demonstrated a survival benefit associated with ICD use in this population as well.^10–14^

Nevertheless, despite their clear use-associated benefits in many situations, ICDs are far from perfect. The potential for numerous complications exists, including infection, vascular occlusions, and lead and/or device malfunction, to name only a few. Additionally, pediatric and some CHD patients have a higher experience rate of many of these complications than healthy adult patients, due in part to the former’s younger age, smaller size, more active lifestyle, growth over time, and anatomic constraints.^11,15^ In particular, pediatric and CHD ICD patients experience inappropriate ICD shocks at a rate much higher than that in other patient groups, with most studies indicating that at least 20% of pediatric and CHD patients experience this phenomenon,^14^ a rate nearly double that observed in some large studies involving adult patients.^18^ This review discusses the importance of addressing inappropriate shocks in pediatric and CHD populations, and presents potential management options for reducing their frequency.

### Implications of inappropriate shocks

**Figure 1:**
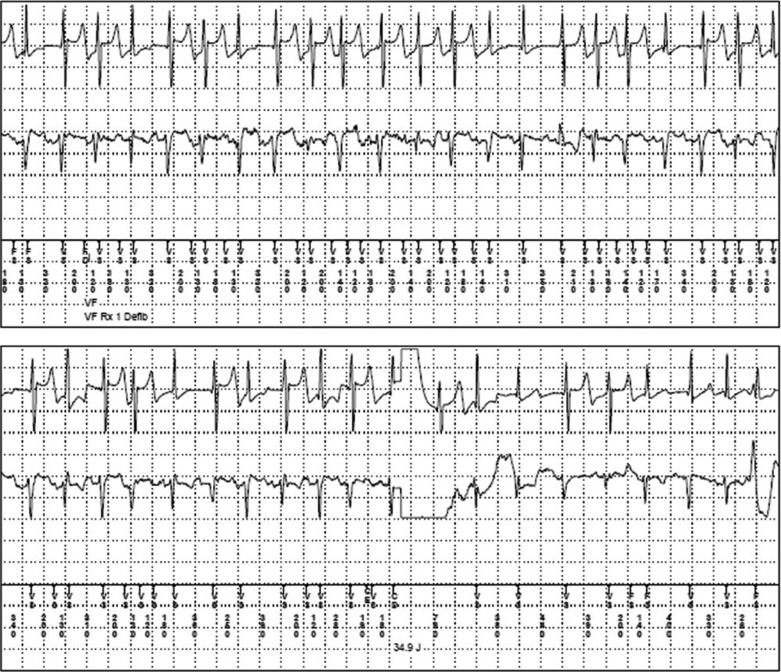
Inappropriate shock as a result of oversensing noise on the RV lead. Top electrogram is RV tip to RV ring; bottom electrogram is can to RV coil.

An inappropriate shock (IS) is an ICD shock delivered for a reason other than for a potentially life-threatening ventricular tachycardia (VT) or ventricular fibrillation (VF) event **(Figure 1).** The causes of inappropriate shocks are variable, and include sinus tachycardia, supraventricular tachycardia (SVT), lead fracture, T-wave oversensing, and external noise. These can be painful and disturbing events for patients to experience. Adult patients receiving an IS have been shown to have increasing perceptions of pain and increased consideration of device inactivation, in comparison to those receiving appropriate shocks alone,^17^ although this correlation has not been clarified in pediatric or CHD patients.^18–21^ An increased number of shocks has also been associated with increased anxiety, depression, and psychological stress.^22–24^ Additionally, the delivery of shocks has a detrimental effect on device battery longevity; an increased number of shocks delivered means a quicker reduction in total battery life, potentially necessitating device replacement at an earlier point in time.

Not only do inappropriate shocks cause painful episodes that adversely affect quality of life, but also they have been associated with negative clinical outcomes. Analyses of the MADIT-II and SCD-HeFT data first demonstrated an independent association between IS and a twofold increase in mortality in adult patients.^16,25^ A similar association has been subsequently demonstrated in other studies,^26,27^ and supported by two recent meta-analyses.^28,29^ The data from these studies conflict, however, with those from another meta-analysis that demonstrated equivocal results, and other recent studies that found no association between IS and mortality.^30,31^

The underlying cause of this assumption has not been completely elucidated. As discussed by Li et al. in their review, it is unclear if inappropriate shocks cause myocardial damage or are pro-arrhythmic in such a way as to contribute directly to this increased mortality, or if they are merely a marker of more significant underlying cardiovascular disease.^32^ The latter explanation is supported by data from the ALTITUDE study, which demonstrated increased mortality in those patients receiving inappropriate shocks for AF or atrial flutter, but not in those patients receiving inappropriate shocks for sinus tachycardia or noise/artifact.^33^

Although inappropriate shocks are a significant problem for ICD patients in general, they are an even more common problem in the pediatric and CHD population. In larger adult studies, the rate of IS has been reported to range from 10% to 15%.^16,25,27^ However, most studies of ICD use in pediatric and CHD patients have found rates of IS to be between 20% and 30% in these patient populations,^10,11,13,14,34–40^ with some studies observing IS in up to 40% to 50% of patients and, in some instances, with inappropriate shocks being more frequent than appropriate shocks.^12,19,41,42^ This is likely a result of a more active lifestyle, faster rates of sinus tachycardia, longer duration of implantation, smaller patient size, and a higher rate of device and lead complications relative to adult patients.^11,15^

Inappropriate shocks are painful events that confer no benefit to patients, are associated with worsened psychosocial and clinical outcomes, and are particularly frequent in the pediatric and CHD ICD populations. Because inappropriate shocks are known to be a significant clinical problem in this population, every effort should be made to reduce their frequency without compromising patient safety.

### Interventions to reduce rates of inappropriate shock

Inappropriate shocks are clearly a significant problem in pediatric and CHD ICD patients, and safely reducing their frequency seems to be an obvious goal. Tools that can help accomplish this can be broken down into two subsets: non-device-based interventions, and device-based interventions.

### Non-device-based interventions

#### Antiarrhythmic medications

Treatment with antiarrhythmic medications has the potential to decrease appropriate shocks by decreasing ventricular arrhythmias, and may also reduce the frequency of inappropriate shocks by reducing supraventricular arrhythmias and blunting maximal rates of sinus tachycardia. A prospective placebo-controlled trial in adult ICD patients demonstrated a reduction in shocks from treatment with sotalol therapy^43^ in comparison with treatment with β-blockers alone.^44^ Data from the MADIT-CRT study found reduced shocks in patients treated with carvedilol versus metoprolol.^45^ Despite this, antiarrhythmic medications are not commonly used in adult ICD patients, with the National Cardiovascular Data Registry ICD registry demonstrating that only 15% of adult ICD patients were discharged on antiarrhythmic medication.^46^

The rate of antiarrhythmic medication use in pediatric and CHD patients appears to be higher than that seen in adult patients. Most studies have found that at least 40%, and up to 100%, of patients are on antiarrhythmic medications, primarily β-blockers.^10,13,39–41,47–49^ This may be a reflection of the different substrates present in the pediatric and CHD population, particularly channelopathy patients, in whom β-blockers are a mainstay of treatment. The use of antiarrhythmic medication has not been conclusively demonstrated to reduce the level of shock burden in this population, despite the common practice of altering medical management in patients who receive shocks.^36^ However, the high baseline rate of antiarrhythmic medication use makes this a difficult association to find in the absence of a randomized trial.

Notably, in the absence of more conclusive data in this population, the use of antiarrhythmic medications, particularly β-blockers, is common practice, and is a reasonable recommendation to include in a shock-reduction protocol.

#### Cardiac ablation

Ablation has long been used as part of shock-reduction protocols to provide targeted elimination of supraventricular or ventricular tachyarrhythmias. The SMASH-VT study found that the ablation of VT in adult patients post-myocardial infarction reduced the number of patients receiving appropriate ICD shocks from 33% to 12%, without increasing mortality.^50^ The VTACH study found that prophylactic VT ablation in adult patients with coronary artery disease reduced appropriate shocks from 47% to 29%, again without increasing mortality.^51^ These studies demonstrate the ability to reduce appropriate shocks by reducing VT substrates in adults with coronary artery disease. Although not specifically evaluated in ICD patients, several studies have demonstrated successful ablation of VT in patients with repaired CHD, predominantly tetralogy of Fallot and Mustard/Senning patients.^52,53^

However, the ability to reduce inappropriate shocks by eliminating SVT substrates has not been well studied, particularly in the pediatric and CHD populations. In the absence of specific data to guide clinical decision-making, for patients with known SVT, or those receiving an IS for SVT, an ablation would seem to be a reasonable approach to reducing inappropriate shock, provided that there are no contraindications to the performance of the procedure.

### Device-based interventions—data from studies in adult patients

Although non-device-based interventions are a useful component of a shock-reduction strategy, appropriate device programming can have a significant impact on shock burden. Early in the experience with ICD programming, focus was placed on delivering shock therapy rapidly. However, over time, it became clear that this aggressive strategy resulted in unnecessary shocks, both appropriate and inappropriate, and a number of ICD programming interventions began to be studied in adult populations in an effort to reduce the shock burden. These interventions primarily fall into three categories: alterations to detection rate or duration programming, use of antitachycardia pacing (ATP), and utilization of discriminator algorithms to more accurately exclude non-ventricular arrhythmias.

#### Alterations in detection rate or duration programming

The most intuitive programming intervention to reduce shock burden is alterations in tachyarrhythmia detection, either by increasing the tachycardia detection rate or the time to tachycardia detection. An early study to evaluate this was the PREPARE study, which prospectively evaluated 700 patients imbued with a specific more lenient programming strategy (including a VT detection rate of 182 bpm), in comparison with patients in the EMPIRIC and MIRACLE ICD trials, which considered more conventional programming. Not surprisingly, the lenient programming resulted in a significant reduction in shocks; however, interestingly, there was also no increase in mortality or morbidity, which had been one of the arguments against a more lenient programming strategy.^54^ A similar result was demonstrated for prolonged detection intervals in the RELEVANT study.^55^ In one of the more significant studies on the issue, MADIT-RIT reported on outcomes using lenient programming strategies. One thousand five hundred primary prevention patients were randomized to one of three programming options: conventional therapy (>170 bpm VT zone with 2.5-s delay to shock, and >200 bpm with a 1.0-s delay); high rate therapy (>200 bpm 2.5-s delay); and delayed therapy (>170 bpm with a 60-s delay, >200 bpm with a 12-s delay, and > 250 bpm with a 2.5-s delay). Both the delayed therapy and high rate therapy programming strategies were associated with decreased rates of appropriate and inappropriate shocks, and with a decrease in all-cause mortality by approximately 50%, with no increase in the rates of syncope.^56,57^ These studies had a significant impact on ICD programming strategies, and spawned a number of similar studies examining variations on lenient programming practices. Several studies have examined high rate programming,^58,59^ while others have considered prolonged detection,^60–65^ or a combination of these two approaches.^66,67^ In general, lenient programming practices in these studies have demonstrated a decrease in the rate of appropriate and inappropriate shocks, with either a decrease in overall mortality or no difference, when compared to conventional programming practices. These practices have been further solidified in two meta-analyses, which found lenient programming practices were associated with a reduction in mortality and IS rates without an increase in syncope.^68,69^ It is clear that in these populations, adopting lenient programming strategies is a safe and effective way to reduce shocks.

#### Use of ATP

**Figure 2:**
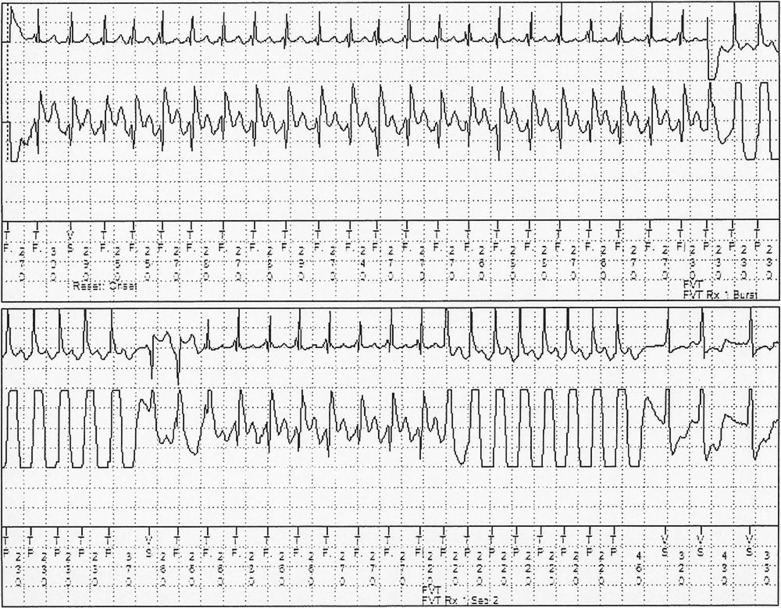
Ventricular tachycardia terminated by the second round of ventricular ATP. Top electrogram is RV tip to RV ring; bottom electrogram is can to RV coil.

In addition to these programming strategies, ATP is also frequently used as part of shock reduction protocols **(Figure 2).** Although the ability to terminate VT using ATP is well known, the use of ATP was minimized in early clinical trials out of safety concerns. In the PainFREE Rx II study, 634 adult patients were randomized to undergo either ATP first or shock first for fast VT detection. ATP was found to be effective in over 70% of episodes, and there was no increase in the rates of syncope, sudden death, or arrhythmia acceleration in the ATP group.^70^ A similar reduction in shock burden without a compromise in safety was demonstrated in the EMPIRIC trial.^71^ These findings suggest that the use of ATP is an important programming component as part of a shock reduction protocol. However, the success of ATP likely depends on the substrate present, and its success rates in younger patients, and in channelopathy patients in particular (who were excluded from the PainFREE Rx II study), have not been well studied. Limited data suggest that two sequences are more effective than one, and that 15 sequences are no better than eight.^72,73^

#### Use of discriminator algorithms

Various programmable discriminators have been utilized to reduce shock burden by attempting to improve identification of sinus tachycardia and SVT that may fall within the programmed VT/VF zones, and withhold therapies. These discriminators are heterogeneous, and details vary depending on the device manufacturer and model. These primarily evaluate either arrhythmia timing (the abruptness of onset, stability, or duration) or the morphology of the electrogram relative to stored templates during sinus rhythm. The primary argument against the use of these options has been concerns that their use would lead to a delay in or the withholding of treatment for true VT/VF. However, the use of discriminators has been shown to decrease the rate of inappropriate shocks without compromising appropriate therapy or increasing mortality as compared with rate-only programming.^74,75^ Nevertheless, relative to other programming parameters, there are fewer studies that directly evaluate the safety and reliability of discriminators, and the variety and heterogeneity of these parameters makes it difficult to determine their optimal use in clinical management.

A subset of these algorithms attempts to use information from the atrial lead in dual-chamber devices to better discriminate between VT/VF and SVT or sinus tachycardia, primarily based on the A/V relationships and timing. Data on the results of these algorithms are mixed, with some studies demonstrating a decrease in the rate of inappropriate shocks in dual-chamber ICD patients,^76^ and others finding no difference.^77^ A meta-analysis on the subject was also equivocal, finding a small reduction in the total number of inappropriate shocks delivered in dual-chamber ICDs, but no reduction in the number of patients experiencing an episode of inappropriate shock.^78^ In the absence of more convincing data, and given the potential for increased costs and complications associated with the presence of an additional lead, it seems that the addition of an atrial lead should be reserved for those patients with an indication for atrial pacing.

### Device-based interventions in pediatric and CHD patients

Data from studies in adult patients have demonstrated that device-based programming strategies can reduce the rates of IS without compromising safety, and a recent consensus statement provides recommendations for ICD programming in the adult population.^79^ However, despite the high rates of IS in pediatric and CHD patients, there are few studies that have been published that evaluate programming practices in this population. A study by Love et al. evaluating 54 ICD patients reported a rate of IS of 15% caused by either SVT or sinus tachycardia. They noted a lower programmed rate for shock therapy in the group that received an IS (186 bpm versus 200 bpm) that was statistically significant. For those patients receiving an IS, a number of interventions were undertaken, including increasing VT detection rate and duration, and increasing the dose of antiarrhythmic medications. According to the study results, none of these patients went on to experience another IS, although the specific details of the programming changes were not reported. The study’s authors also noted the rare use of SVT discriminator algorithms as an institutional practice at the time. They conclude that setting a ventricular detection rate above the maximum expected sinus rate may be a reasonable approach to minimizing the rate of IS.^80^ In 2015, we published a single-center retrospective review of 144 patients, and reported a rate of IS under 10% using a general institutional practice of programming relatively high detection rates and long detection durations. Specifically, mean tachycardia detection rate was 222 bpm, and mean detection duration was 18 beats. Although patients with shocks programmed in a VT zone were more likely to experience an IS, an association with detection rates or durations and IS could not be made, given that patients were programmed fairly similarly. No episodes of syncope or undertreated VT/VF were identified.^48^ The practice of programming higher detection rates would seem consistent with data that have demonstrated higher detected ventricular tachycardia rates in the CHD population relative to the general adult population. Khairy et al. reported data from tetralogy of Fallot and transposition of the great arteries patient populations, demonstrating detection rates in patients receiving an appropriate shock of 213 bpm and 222 bpm, respectively.^34,81^ Additionally, in a population of ICD patients with Brugada syndrome, the mean detected tachycardia rate in appropriate shocks was 335 bpm, with the suggestion that a single VF zone at 222 bpm would reduce IS by 70%, although it could potentially lead to the missing of appropriate shocks in 1.7% of patients.^82^

The frequency of use of ATP and its effectiveness in this population is not well known, although some authors have found the success rate to be low, and have advocated against its routine use.^37^ However, in a single-center study of 79 pediatric and CHD patients, ATP was successful at terminating VT in 88% of episodes, with 85% of episodes being terminated within the first ATP attempt. Acceleration of the VT with ATP was seen in three episodes (3%), which either spontaneously terminated, were terminated by additional ATP, or were converted by a shock in one case each. ATP was similarly effective in patients with CHD and cardiomyopathy, but not effective in patients with primary electrical disease. One episode of proarrhythmia from inappropriate ATP was reported, which was terminated with a shock.^43^

The utility of dual-chamber discriminator algorithms to reduce the rates of IS has been investigated in pediatric and CHD patients. In a multi-center study by Lawrence et al. of 168 ICD patients, 21% of whom received an IS, there was no difference in the rate of IS or appropriate shocks between single- and dual-chamber devices, suggesting that the presence of an atrial lead does not enhance rhythm discrimination in this population, a finding that was supported by our single-center report as well.^48,83^ These data again support the notion that the addition of an atrial lead should be reserved for those patients with a specific pacing indication, rather than for instances of enhanced rhythm discrimination alone.

## Summary of programming recommendations

In the absence of a significant body of published literature to guide programming decisions in the pediatric and CHD ICD populations, clinicians are left to (1) interpret what we can from the limited published data, (2) extrapolate with caution from the literature on adult patients, and (3) rely on clinical experience and expert recommendations.^84^ As is true for many aspects of caring for this heterogeneous population, a “one-size-fits-all” approach is overly simplistic, and preset device “nominal” settings (based on the typical adult patient) are often inappropriate. Programming practices must be individualized to the specific patient and clinical situation. However, certain general recommendations can be made that are applicable in many—though not all—situations, with the goal of safely reducing the rate of IS. **Table 1** provides some considerations to keep in mind when assessing and working with a pediatric or CHD ICD patient.

## Conclusions

ICDs are effective devices for use in treating pediatric and CHD patients at risk for life-threatening ventricular arrhythmias. However, the rates of device complications in these individuals can be excessive, with inappropriate shocks in particular representing a significant problem. There are a number of both non-device-based and device-based interventions that have the potential to significantly decrease the rates of IS in these patient populations, without compromising safety. Additional data are needed to evaluate the outcomes of these strategies in real-world settings in pediatric and CHD ICD patients.

## Figures and Tables

**Table 1:** Pediatric and CHD ICD Programming Considerations

1.	Individualize programming	Programming should be individualized for each patient. The use of pre-programmed “nominal” settings is rarely appropriate.
2.	Be proactive	Consider implementing programming strategies aimed at decreasing inappropriate shocks at the time of implant, rather than waiting for an IS to happen in order to adopt these strategies.
3.	Program faster detection rates	If rates of spontaneously hemodynamically significant VT/VF are known, detection should be programmed below this rate. In the absence of known slow VT, programming therapy should be avoided at rates below the expected maximal sinus rate, at least 200 bpm in most patients and > 220 bpm in many patients, especially those who are younger.
4.	Program longer detection times	Perform this at a minimum of 18/24 beats in most patients, with strong consideration of 24/32 or 30/40 intervals.
5.	Utilize ATP where applicable	ATP appears to be minimally effective in most channelopathy patients, in whom ventricular arrhythmias are not typically pace-terminable, and should therefore be avoided. In patients with cardiomyopathies or CHD, ATP should be programmed either before or, at minimum, during charging. At least two sequences of ATP should be employed in most situations.
6.	Utilize SVT discriminators when appropriate	Deactivate in patients with AV block, who cannot conduct SVT rapidly. Otherwise, the use of discriminators is reasonable, especially if therapies are programmed on for slower zones (ie, > 220 bpm for younger patients, > 200 bpm in older patients and adults). If used, the “time out” functions should be disabled, to prevent the delivery of therapy for a prolonged SVT episode.
7.	Implant single chamber devices as default	The addition of an atrial lead should be reserved only for patients with an indication for a dual-chamber system, and not for the purpose of improving tachycardia detection alone.
8.	Encourage antiarrhythmic medications	Do this especially with b-blockers, which can reduce IS by reducing rates of sinus tachycardia and the incidence of SVT.
9.	Consider SVT ablation	Contemplate this in patients with a known history of SVT (depending on underlying anatomical considerations), or for patients who receive an IS for SVT, especially if it is not controlled with medications.
